# Impact of Cyanidin-3-Glucoside on Glycated LDL-Induced NADPH Oxidase Activation, Mitochondrial Dysfunction and Cell Viability in Cultured Vascular Endothelial Cells

**DOI:** 10.3390/ijms131215867

**Published:** 2012-11-27

**Authors:** Xueping Xie, Ruozhi Zhao, Garry X. Shen

**Affiliations:** Departments of Internal Medicine and Physiology, University of Manitoba, 835-715 McDermot Ave, Winnipeg, MB R3E 3P4, Canada; E-Mails: xiexp@yahoo.com (X.X.); zhaor@cc.umanitoba.ca (R.Z.)

**Keywords:** cyanidin-3-glucoside, glycated LDL, NADPH oxidase, endothelial cells, mitochondrial electron transport chain enzymes, cell viability

## Abstract

Elevated levels of glycated low density lipoprotein (glyLDL) are frequently detected in diabetic patients. Previous studies demonstrated that glyLDL increased the production of reactive oxygen species (ROS), activated NADPH oxidase (NOX) and suppressed mitochondrial electron transport chain (mETC) enzyme activities in vascular endothelial cells (EC). The present study examined the effects of cyanidin-3-glucoside (C3G), a type of anthocyanin abundant in dark-skinned berries, on glyLDL-induced ROS production, NOX activation and mETC enzyme activity in porcine aortic EC (PAEC). Co-treatment of C3G prevented glyLDL-induced upregulation of NOX4 and intracellular superoxide production in EC. C3G normalized glyLDL-induced inhibition on the enzyme activities of mETC Complex I and III, as well as the abundances of NADH dehydrogenase 1 in Complex I and cytochrome b in Complex III in EC. Blocking antibody for the receptor of advanced glycation end products (RAGE) prevented glyLDL-induced changes in NOX and mETC enzymes. Combination of C3G and RAGE antibody did not significantly enhance glyLDL-induced inhibition of NOX or mETC enzymes. C3G reduced glyLDL-induced RAGE expression with the presence of RAGE antibody. C3G prevented prolonged incubation with the glyLDL-induced decrease in cell viability and the imbalance between key regulators for cell viability (cleaved caspase 3 and B cell Lyphoma-2) in EC. The findings suggest that RAGE plays an important role in glyLDL-induced oxidative stress in vascular EC. C3G may prevent glyLDL-induced NOX activation, the impairment of mETC enzymes and cell viability in cultured vascular EC.

## 1. Introduction

Hyperglycemia and dyslipoproteinemia are two major biochemical markers of diabetes. Increased low density lipoprotein (LDL) is a classical risk factor for atherosclerotic cardiovascular disease. Elevated levels of glycated LDL (glyLDL) were frequently detected in type 1 or type 2 diabetic patients [1,2]. Endothelium is a cellular monolayer between blood components and other vascular tissues. Active interactions between glyLDL and vascular endothelial cells (EC) may play important roles in the acceleration of atherosclerotic cardiovascular disease in diabetic patients. GlyLDL stimulated the generation of reactive oxygen species (ROS) in EC [3]. NADPH oxidase (NOX) and mitochondria are two major sources of ROS in EC. NOX is a group of intracellular enzymes and mediates the generation of superoxide from NADPH [4]. NOX4 was detected at the mitochondrial inner membrane. The activation of NOX4 may affect mitochondrial activity through ROS formation [5]. Mitochondria produce ROS during electron transport. Mitochondrial dysfunction may lead to excess electron leak, superoxide production and mitochondria-dependent apoptosis [6]. Recent studies in our laboratory demonstrated that glyLDL increased NOX2 abundance, reduced activities of multiple mitochondrial electron transport chain (mETC) enzymes and increased the abundance of mitochondria-associated ROS in aortic EC [7].

Anthocyanidins are a group of plant flavanoids abundant in dark-skinned fruits, as well as in vegetables or grains. Previous studies demonstrated that dark-skinned berries possess antioxidant properties in cell culture or animal models [8,9]. Cyanidin-3-glucoside (C3G) is one of most common forms of anthocyanins (glycans of anthocyanidins) abundant in the blueberry, Saskatoon berry, raspberry, strawberry and chokecherry [10]. C3G blocked ethanol-induced intracellular accumulation of ROS in neural cells [11]. The effect of C3G on glyLDL-induced NOX4 expression, mitochondrial dysfunction or apoptosis in EC remains unclear.

The present study examined the effects of C3G on glyLDL-induced ROS production, RAGE, NOX4 content, mETC enzyme activity and cell viability in cultured porcine aortic EC (PAEC).

## 2. Results and Discussion

### 2.1. Effect of C3G on GlyLDL-Induced ROS Production

**Figure 1:**
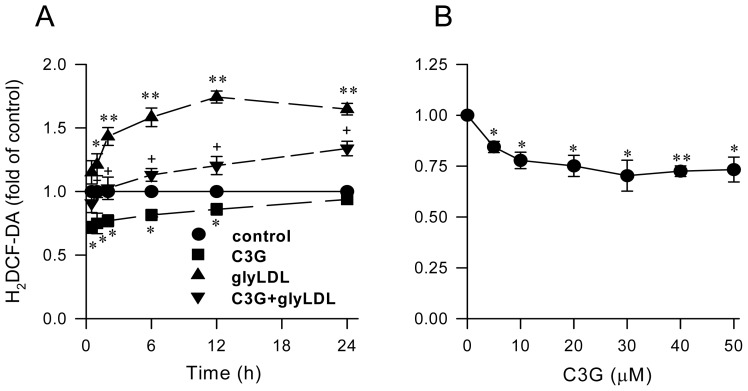
Effect of C3G on redox status in EC. PAEC were treated with vehicle (control), 30 μM C3G, 100 μg/mL of glyLDL or C3G + glyLDL for 0.5–24 h (**A**) or with 0–50 μM C3G for 30 min (**B**). Redox status was assessed by measuring fluorescence intensity in cells at 485/530 nm (excitation/emission) using a fluorescence microplate reader. Values were expressed in mean ± SD in fold of control (*n* = 3 independent experiments). *, **: *p* < 0.05 or 0.01 *versus* control; +: *p* < 0.05 *versus* glyLDL.

The effect of C3G on intracellular redox status was characterized in PAEC by assessing the level of H_2_DCF-DA. GlyLDL (100 μg/mL for 1–24 h) significantly increased the fluorescent intensity of H_2_DCF-DA in PAEC (*p* < 0.05 or 0.01). Co-treatment with 30 μM C3G significantly reduced glyLDL-induced increases in redox status in PAEC (*p* < 0.05 or 0.01). The maximal inhibition of C3G on glyLDL-induced redox status detected in EC treated with glyLDL and C3G for 12 h. Significant inhibitions on redox status were detected in PAEC treated with 30 μM C3G for 10 min to 12 h (Figure 1A). Since the peak inhibition of C3G alone on redox was found in PAEC treated with 30 μM C3G for 30 min (Figure 1B), a pretreatment with 30 μM C3G was applied in experiments with or without addition of lipoproteins in this study.

**Figure 2:**
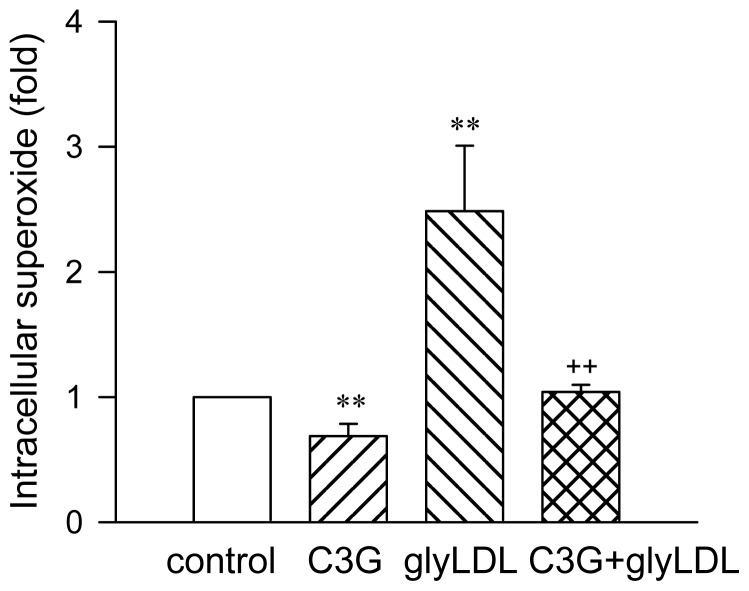
Effect of C3G on glyLDL-induced intracellular superoxide in EC. PAEC were treated with vehicle (control), C3G (30 μM), glyLDL (100 μg/mL) or C3G + gLDL for 2 h. Intracellular superoxide was measured using lucigenin method. Values were expressed in means ± SD in fold of control (*n* = 3 independent experiments). **: *p* < 0.01 *versus* control; ++: *p* < 0.01 *versus* glyLDL.

Treatment with 100 μg/mL of glyLDL for 2 h increased the levels of intracellular superoxide in PAEC by more than two-folds (*p* < 0.01). C3G alone at 30 μM reduced intracellular superoxide compared to control (*p* < 0.01). Co-treatment of C3G blocked glyLDL-induced increase of intracellular superoxide in EC compared to glyLDL alone (*p* < 0.01, Figure 2).

### 2.2. Effects of C3G on the Activities of mETC Enzymes in GlyLDL-Treated EC

**Figure 3:**
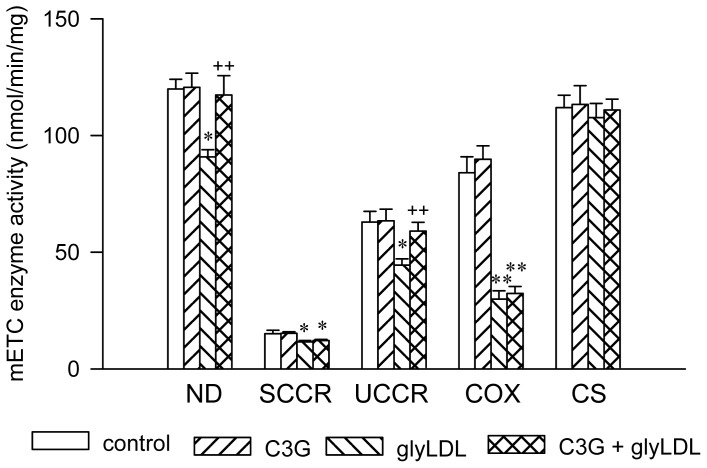
Effects of C3G on the activities of mitochondrial respiratory chain complex enzymes in glyLDL-treated EC. PAEC were treated with vehicle (control), 30 μM C3G, 100 μg/mL of glyLDL or C3G + glyLDL for 12 h. Activities of NADH dehydrogenase (ND), succinate cytochrome c redutase (SCCR), ubiquinol cytochrome c reductase (UCCR), cytochrome c oxidase (COX) and citrate synthase (CS) in EC were analyzed as described in the experimental section. Values were expressed in mean ± SD in nmol/min/mg protein (*n* = 3 independent experiments). *, **: *p* < 0.05 or 0.01 *versus* control; ++: *p* < 0.01 *versus* glyLDL.

Our previous studies demonstrated that glyLDL (100 μg/mL) significantly decreased the activities of ND and SCCR in PAEC after ≥12 h of incubation, and that of UCCR or COX after ≥6 h of incubation [12]. The present study assessed the effects of treatment with 30 μM C3G for 12 h on the activities of ND, SCCR, UCCR and COX in PAEC in the presence or absence of 100 μg/mL of glyLDL, and compared to the activity of CS (a control for mitochondrial enzyme). Treatment with glyLDL significantly decreased the activities of ND, SCCR, UCCR and COX compared to vehicle control (*p* < 0.05 or 0.01), but did not affect the activity of CS in PAEC. Co-treatment with C3G normalized glyLDL-induced reduction in ND and UCCR activities compared to EC treated with glyLDL alone (*p* < 0.01), but did not significantly alter the levels of CS, SCCR or COX activity (Figure 3).

### 2.3. Effects of C3G on GlyLDL-Induced Changes in NOX4, ND1 and Cyt b

**Figure 4:**
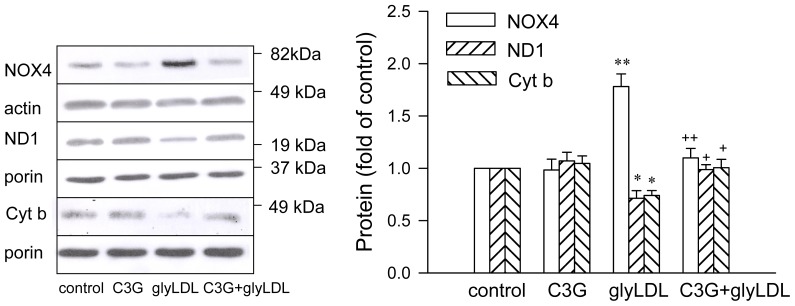
Effects of C3G on glyLDL-induced changes in NOX4, ND1 and Cyt b content. PAEC were treated with vehicle (control), 30 μM C3G, 100 μg/mL of glyLDL or C3G + glyLDL for 12 h. Abundances of NOX4, ND1 and Cyt b were measured using Western blotting. Molecular weights of standards were marked beside the blots. Values were expressed in mean ± SD folds of control (*n* = 3 independent experiments). *, **: *p* < 0.05 or 0.01 *versus* control; +, ++: *p* < 0.05 or 0.01 *versus* glyLDL.

Treatment with glyLDL at 100 μg/mL for 12 h significantly increased the abundance of NOX4, and reduced levels of ND1 (a subunit of Complex I enzyme) and cytochrome b (Cyt b, a subunit of Complex III enzyme) in PAEC after normalization with the levels of β-actin or porin in corresponding samples (*p* < 0.05 or 0.01). C3G (30 μM) alone did not significantly alter the level of NOX4, ND1 or Cyt b in EC. Co-treatment with C3G normalized glyLDL-induced changes in NOX4, ND1 and Cyt b in EC (*p* < 0.05 or 0.01, Figure 4).

### 2.4. Effect of RAGE Blockage and C3G on GlyLDL-Induced Changes in RAGE, NOX and mETC Enzymes

**Figure 5:**
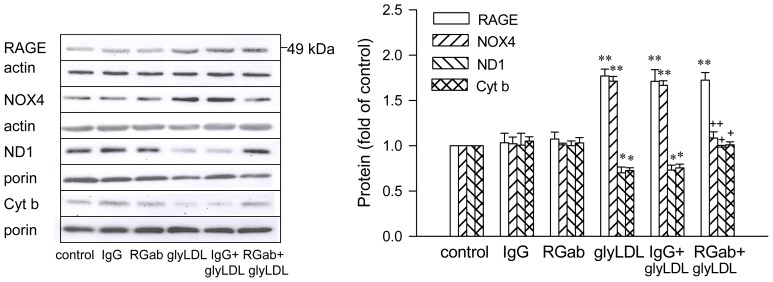
Effects of RAGE antibody on glyLDL-induced changes in RAGE, NOX4, ND1 and Cyt b content. PAEC were treated with vehicle (control), 10 μg/mL of rabbit IgG, 10 μg/mL of RAGE antibody (RGab), 100 μg/mL of glyLDL, IgG + glyLDL or RGab + glyLDL for 12 h. Abundances of NOX4, RAGE, ND1 and Cyt b were measured using Western blotting. Values were expressed in mean ± SD folds of control (*n* = 3 independent experiments). *, **: *p* < 0.05 or 0.01 *versus* control; +, ++: *p* < 0.05 or 0.01 *versus* glyLDL.

**Figure 6:**
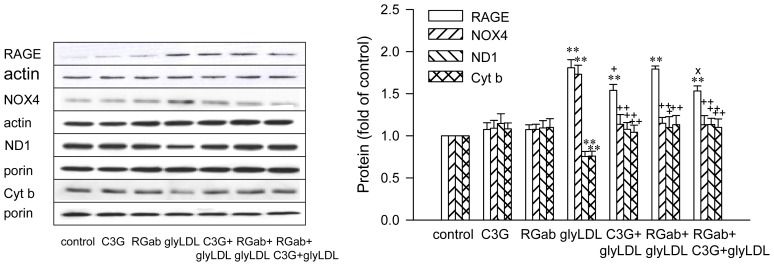
Effects of C3G and RAGE antibody on glyLDL-induced changes in RAGE, NOX4, ND1 and Cyt b content. PAEC were treated with vehicle (control), 30 μM C3G, 10 μg/mL of RAGE antibody (RGab), 100 μg/mL of glyLDL, C3G + glyLDL or RGab + C3G + glyLDL for 12 h. Abundances of NOX4, RAGE, ND1 and Cyt b were measured using Western blotting. Values were expressed in mean ± SD folds of control (n = 3 independent experiments). **: *p* < 0.01 *versus* control; +, ++: *p* < 0.05 or 0.01 *versus* glyLDL; x: *p* < 0.05 *versus* glyLDL plus RGab.

Blocking antibody for RAGE alone or rabbit IgG did not significantly alter the levels of RAGE, NOX4, ND1 or Cyt b in EC. Co-treatment with RAGE antibody, but not IgG, prevented glyLDL-induced increase in NOX4 and the decrease in the abundance of ND1 or Cyt b in PAEC. (*p* < 0.05 or 0.01, Figure 5). RAGE antibody or IgG did not significantly affect glyLDL-induced increase of abundance of RAGE. Co-treatment with C3G or RAGE antibody normalized the changes in the contents of NOX4, ND1 and Cyt b induced by glyLDL in PAEC (*p* < 0.01), but did not significantly affect the level of RAGE in PAEC. Co-treatment of C3G with RAGE antibody did not show an additive effect on glyLDL-induced changes in NOX4, ND1 or Cyt b in PAEC compared to glyLDL-treated EC with the addition of C3G or RAGE antibody alone. The addition of C3G significantly reduced glyLDL-induced increase in abundance of RAGE compared to PAEC treated with or without the presence of RAGE antibody (Figure 6).

### 2.5. Effect of GlyLDL on Cell Viability

**Figure 7:**
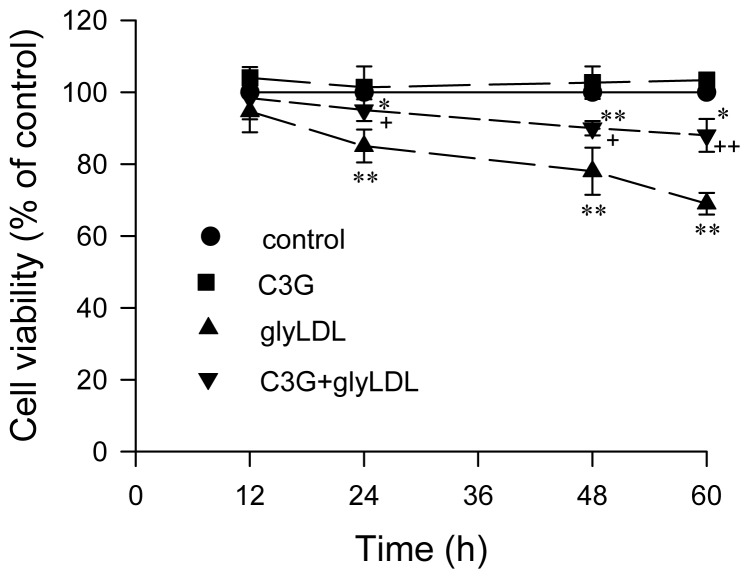
Effects of C3G and glyLDL on cell viability. PAEC were treated with vehicle (control), 30 μM C3G, 100 μg/mL of glyLDL or C3G + glyLDL for 12–60 h. Cell viability was measured using MTT assay as described in the experimental section. Values were expressed in means ± SD in percentage of control (*n* = 3 independent experiments). *, **: *p* < 0.05 or 0.01 *versus* control with corresponding incubation time; +, ++: *p* < 0.05 or 0.01 *versus* glyLDL.

The effect of glyLDL on cell viability was examined in PAEC treated with 100 μg/mL of glyLDL for 12–60 h using (3-(4,5-di-methylthiazol-2-yl)-2,5-diphenyltetrazolium bromide (MTT) assay. The cell viability of PAEC was significantly decreased by treatment with 100 μg/mL of glyLDL for ≥24 h (*p* < 0.01), but not in cells treated with glyLDL for 12 h. Treatment with 30 μM C3G prevented glyLDL-induced impairment of cell viability after 24 h, 48 h or 60 h compared to EC exposed to glyLDL alone (*p* < 0.05 or 0.01, Figure 7).

### 2.6. Effects of GlyLDL on Regulators for Cell Viability

**Figure 8:**
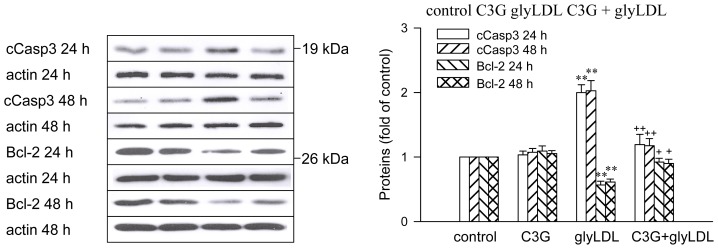
Effects of C3G and glyLDL on regulators for cell viability. PAEC were treated with vehicle (control), 30 μM C3G, 100 μg/mL of glyLDL or C3G + glyLDL for 24 h or 48 h. Abundances of cleaved caspase 3 (cCasp3) and Bcl-2 were measured using Western blotting. Values were expressed in mean ± SD folds of control (*n* = 3 independent experiments). **: *p* < 0.01 *versus* control; +, ++: *p* < 0.05 or 0.01 *versus* glyLDL.

GlyLDL treatment (100 μg/mL) for 24 h or 48 h significantly increased the abundance of cleaved caspase 3, a mitochondria-dependent apoptosis agonist, and decreased the abundance of Bcl-2, a negative regulator for apoptosis, in PAEC compared to controls (*p* < 0.01). No significant difference in cleaved caspase 3 or Bcl-2 was detected between EC treated with glyLDL for 24 h and 48 h. Co-treatment with 30 μM C3G normalized glyLDL-induced changes in cleaved caspase 3 and Bcl-2 in PAEC compared to that treated with glyLDL alone (*p* < 0.05 or 0.01). C3G alone did not significantly alter the level of cleaved caspase 3 or Bcl-2 in PAEC (Figure 8).

### 2.7. Discussion

The present study provided the following novel findings regarding the protective effects of C3G on cellular events related to diabetic vascular injury: (1) C3G reduced ROS production in PAEC at basal and glyLDL-treated condition; (2) C3G normalized glyLDL-induced attenuation in the activities of mETC Complex I and III enzymes and reduced abundances of ND1 and Cyt b in the complexes in PAEC; (3) C3G or RAGE antibody blocked glyLDL-induced increase of NOX4 and decreased in ND1 and Cyt b, and C3G reduced glyLDL-induced increase in the abundance of RAGE in PAEC; (4) C3G prevented glyLDL-induced decrease cell viability and the imbalance between cleaved caspase 3 and Bcl-2 in PAEC.

Previous studies demonstrated that anthocyanidins reduced susceptibility of vascular EC to ROS and tumor necrosis factor (TNF)-α [13]. Artisotelia chileansis berry juice decreased intracellular oxidative stress induced by H_2_O_2_ in cultured EC [14]. Grape seed supplementation protected cardiac function *via* inhibiting lipid peroxidation and apoptosis signaling [15]. C3G attenuated H_2_O_2_- or TNF-α-induced insulin resistance by inhibiting c-Jun NH_2_-terminal kinase activation in 3T3-L1 adipocytes [16]. Our previous studies demonstrated that glyLDL increased the generation of ROS and altered the activities of antioxidant enzymes in vascular EC [3]. GlyLDL treatment significantly impaired oxygen consumption in mETC Complexes I-IV in EC compared to LDL or vehicle control [7]. The results on the present study demonstrated that C3G normalized glyLDL-induced the upregulation of ROS or NOX4, and the attenuation of mETC Complex I and III enzymes in EC. The findings suggest that C3G may modulate intracellular ROS production through inhibiting NOX4 activity and upregulating mETC enzymes in EC at basal or glyLDL-treated condition, which may help to reduce oxidative stress in vascular EC in a diabetic condition.

Arevious study in our laboratory indicated that glyLDL elevated the abundance of NOX2 in human umbilical vein EC and the level of NOX4 was increased in hearts of streptozotocin-induced diabetic mice [17]. The results of the present study demonstrated that C3G not only prevented mitochondrial dysfunction, but also reduced glyLDL-induced elevation in NOX4 abundance and NADPH-dependent intracellular superoxide in PAEC. Since mitochondria contain NOX4, the upregulation of NOX4 may directly aggravate oxidative stress in mitochondria, which may cause mitochondrial dysfunction. The protective effect of C3G on mitochondrial mETC enzymes and their activities may be, at least in part, due to the inhibitory effect of C3G on NOX4 in EC. PAECs have been used in the present study due to the similarity of cardiovascular physiology and their rapid growth, which meets the requirement of a large amount of cells in mitochondrial activity assays, particularly for the Complex I activity assay.

ND, the enzyme of mETC Complex I, mediates the transfer of an electron from NADH to coenzyme Q. ND complex is composed of over 40 subunits of proteins. Among them, only ND1-6 subunits are encoded by mitochondrial DNA [18]. UCCR, the Complex III enzyme, is another important source of ROS in mitochondria. It is composed of 11 subunits, but only one of them, Cyt b, is encoded by mitochondrial DNA. Previous studies indicated that hyperglycemia or diabetes were associated with reduced Complex III enzyme activity [19]. Mitochondrial DNA locates in the mitochondrial matrix and attaches to the inner membrane. Mitochondrial DNA is relatively susceptible to attack by ROS in the mitochondrial matrix partially due to its lack of the protection of histone compared to nuclear DNA [20]. The findings of the present study demonstrated that C3G neutralized the inhibitory effect of glyLDL on the activities of Complex I and III, but not that of Complexes II and IV. In order to determine the mechanism for the beneficial effect of C3G on mitochondrial function, we examined the effects of C3G on the abundance of two mitochondrial DNA-encoded mETC enzyme components, ND1 and Cyt b, in EC. The results indicated that C3G prevented the inhibitory effect of glyLDL on the abundances of ND1 and Cyt b in EC, which suggests that the effect of C3G on mitochondrial ROS in EC may result from the protection of mETC Complex I and III enzyme components.

RAGE is the primary receptor for advanced glycation end products (AGEs) and it mediates the transmembrane signaling of AGEs in many types of cells [21]. The expression of RAGE is low at basal condition and may be upregulated under metabolic stress. Our previous study demonstrated that glyLDL increased expression of RAGE in the human umbilical vein or coronary artery EC, and the RAGE antibody blocked the stimulatory effect of glyLDL on plasminogen activator inhibitor-1 [17]. The present study demonstrated that RAGE antibody prevented glyLDL-induced increase in NOX4 and the decreases in ND1 or Cyt b in PAEC. The modulatory effects of C3G or RAGE antibody on glyLDL-induced NOX4 and mETC enzyme subunits were not superimposed, which suggests that the protective effects of C3G on glyLDL-induced changes in NOX4 or mitochondrial enzymes in EC are mediated through RAGE. C3G, but not RAGE antibody, inhibited glyLDL-induced increase in the abundance of RAGE in PAEC, which suggests the protective effects of C3G may be partially mediated by the inhibition of RAGE in glyLDL-treated EC.

Mitochondrial dysfunction may cause programmed cell death or apoptosis due to increased ROS production and decreased ATP formation. Reduced mETC activity may increase electron leak and ROS formation, and reduce ATP generation [6]. Cytochrome c (Cyt c) is a nuclear DNA encoded mitochondrial protein and functions in electron transport, oxidative phosphoration, redox coupling and ROS scavenging. At a normal condition, Cyt c locates in intermembrane space and binds to cardiolipin in the inner membrane of mitochondria. The oxidation of cardiolipin reduces its binding affinity to Cyt c. Translocation of Cyt c from mitochondria to cytosol and its binding to apoptotic protease activating factor-1 in cytosol plays key roles in caspase-mediated apoptosis [22]. Bcl-2 is a prosurvival protein and binds to membrane of mitochondria or endoplasmic reticulum, which functions as a switch for cell survival or death [23]. The present study demonstrated that prolonged exposure of EC to glyLDL impaired cell viability, increased the content of cleaved caspase 3, and reduced the abundance of Bcl-2 in EC. C3G prevented glyLDL-induced reduction of cell viability and imbalance between cleaved caspase-3 and Bcl-2 in EC, which may be secondary to the inhibitory effect of C3G on glyLDL-induced NOX activation, oxidative stress, ROS production and mitochondrial dysfunction.

## 3. Experimental Sections

### 3.1. Isolation and Modification of Lipoproteins

LDL (density 1.019–1.063) was isolated from the plasma of healthy donors using sequential density floatation ultracentrifugation. LDL was oxidized through dialysis against 5 μM CuSO_4_ for 24 h at 22 °C [12]. LDL was glycated using 50 mM glucose, 50 mM sodium cyanoborohydride and 2 mM EDTA at 37 °C for 2 weeks [24]. The glycation of LDL was assessed using trinitrobenzenesulfonic acid assay as previously described [25]. Lipoproteins were stored in sealed tubes under a layer of nitrogen at 4 °C in dark to prevent auto-oxidation.

### 3.2. Cell Culture

PAEC were obtained from Dr. P.E. DiCorleto in the Cleveland Clinic Research Institute. Cells were grown in Dulbecco’s modified Eagle medium supplemented with 10% fetal bovine serum and 1% penicillin-streptomycin (Invitrogen, Burlington, Canada) [7]. PAEC were chosen for the experiments due to the similarity of swine and human in cardiovascular physiology [26] and the capacity to grow a large amount of cells in culture.

### 3.3. Experimental Incubation

C3G (Polyphenols Laboratories, Sandnes, Norway) was dissolved in 0.01% HCl as instructed by manufacturer. C3G and its solution were handled without direct exposure to light. For experiments requiring treatments with multiple reagents, cells were pretreated with indicated concentrations of C3G, blocking polyclonal antibodies for the receptor of advanced glycation end products (RAGE, provided by Dr. A.M. Schmidt, New York, USA), or vehicle for 30 min at 37 °C under 5% CO_2_ before the addition of lipoproteins or vehicles.

### 3.4. Assessment of Redox Status

Intracellular redox status was assessed using 2,7-dichlorodihydrofluorescein diacetate (H_2_DCF-DA, Molecular Probes, Eugene, OR, USA) assay to avoid colorimetric interference of anthocyanin [27]. PAEC were seeded into 96-well plates (2 × 10^5^ cells/well) and allowed to grow for 24 h. After treatment with C3G and/or lipoproteins, cells were incubated with 20 μM H_2_DCF-DA in HEPES buffered salt solution (HBSS, pH 7.4) for 30 min. At the end of incubation, medium was removed and cells were washed with HBSS 3 times. Intensities of fluorescence in cells were measured at 485/530 nm (excitation/emission) using a fluorescence microplate reader (FLUOStar OPTIMA, BMG Lab Technologies, GmbH, Germany) [28]. Relative redox status in EC was expressed as percentage of control.

### 3.5. Intracellular Superoxide Measurement

Intracellular superoxide was measured using enhanced lucigenin assay as previously described [29]. After experimental incubation, cells were homogenized in lysis buffer (50 mM KH_2_PO_4_, pH 7.0, 1 mM EGTA, 10 μg/mL of aprotinin, 0.5 μg/mL of leupeptin, 1 μg/mL of pepstatin and 0.5 mM phenylmethanesulfonylfluoride). Aliquots of 50 μg of cellular proteins were added in an assay buffer (50 mM KH_2_PO_4_, pH 7.0, 1 mM EGTA, 150 mM sucrose, and 100 μM NADPH). The reaction was started by the addition of 25 μM lucigenin. The levels of NADPH-dependent superoxide was assessed according to chemoluminescence in tested samples detected using a photon counter (Lumat LB 9507, Berthold, Nashua, NH) for 30 min [30].

### 3.6. Western Blotting Assay

Equal amounts of cellular lysate were analyzed with 12% SDS-PAGE and electrotransferred to nitrocellulose membrane. Proteins were identified using antibodies against reduced nicotinamide adenine dinucleotide (NADH) dehydrogenase (ND)1, cytochrome b (Cyt b), NOX4, rabbit IgG (Santa Cruz, CA, USA), β-actin (Sigma, St Louis, MO), RAGE, porin (a control for mitochondrial proteins, Abcam, Cambridge, MA, USA), B cell lymphoma-2 (Bcl-2) or cleaved caspase 3 (Cell Signaling, Pickering, ON, Canada). Enhanced chemiluminescence reagents (Amersham, Piscataway, NJ, USA) were used for detecting targeted antigens on membrane. Densities of antigens were analyzed using Chemi-Doc system with Quantity-One software (Bio-Rad, Hercules, CA, USA). Abundances of targeted proteins were normalized with the levels of control protein (porin for mitochondrial proteins, β-actin for nonmitochondrial cellular proteins) as previously described [7].

### 3.7. ND Activity

ND (Complex I enzyme) activity was measured as described previously [31]. Mitochondrial fractions of PAEC (50 μg) were added to buffer containing 25 mM potassium phosphate (pH 7.2), 5 mM MgCl_2_, 2 mM KCN, 2.5 mg/mL of bovine serum albumin (fraction V), 2 μg/mL of antimycin A, 0.1 mM NADH, and 50 μM decylubiquinone. The measurement of ND activity was started at 3 min before the addition of rotenone (2 μg/mL) and continued for another 3 min at 340 nm using an Ultrospec 2000 UV-visible spectrophotometer equipped with Biochrom Swift II software (Biopharmacia Biotech, Uppsala, Sweden) [32].

### 3.8. Succinate Cytochrome C Reductase (SCCR) Activity

SCCR (Complex II/III enzymes) activity was measured by monitoring the rate of reduced Cyt c formation using succinate as substrate. Sonicated cell lysates (0.2 mg of protein) were pre-incubated with a reaction mixture (10 mM potassium phosphate, pH 7.4, 2 mM EDTA, 0.01% bovine serum albumin, 0.2 mM ATP, 1 mM KCN, 5 μM rotenone, and 10 mM succinate) for 3 min and reaction was started by the addition of 40 μM oxidized Cyt c. Changes in absorbance were monitored at 30 °C using a spectrophotometer for 5 min at 550 nm [33].

### 3.9. Ubiquinol Cytochrome C Reductase (UCCR) Activity

The activity of UCCR (Complex III enzyme) was evaluated using 100 μg of cell lysates with a reaction mixture containing 25 mM potassium phosphate (pH 7.4), 5 mM MgCl_2_, 2 mM KCN, 2 μg/mL of rotenone, 2.5 mg/mL of bovine serum albumin, and 50 μM Cyt c in a final volume of 1 mL. After a 2 min equilibration period, reaction was started by the addition of 50 μM ubiquinol-2. Changes in absorbance at 550 nm were monitored [34].

### 3.10. Cytochrome C Oxidase (COX) Activity

COX (Complex IV enzyme) activity was examined at 30 °C by measuring the rate of oxidation of reduced Cyt c at 550 nm. Assay was performed in the presence of 40 μM reduced Cyt c, 20 mM phosphate buffer, 0.1 mg of protein from PAEC and 16 mg of lauryl maltoside/mg protein (0.16%) [35].

### 3.11. Citrate Synthase (CS) Activity

CS activity was determined at 30 °C in a medium containing 150 mM Tris-HCl (pH 8.2), 0.16% lauryl maltoside, 0.1 mM dithionitrobenzoic acid and 0.1 mg of cellular protein from EC. The reaction was started by adding 300 μM acetyl-CoA. Changes in absorbance were measured at 412 nm for 1 min. The rate of absorbance change was subtracted from that with the addition of 0.5 mM oxalacetic acid. CS activity was used as a control of mitochondrial enzymes [36].

### 3.12. Cell Viability Assay

Cell viability was determined using 3-(4,5-dimethylthiazol-2-yl)-2,5-diphenyltetrazolium bromide (MTT) colorimetric assay. PAEC in 96-well plates (1 × 10^4^/well) cultured to 70%–80% of confluence, and then incubated with indicated agents for 12–60 h. Media were replaced by fresh medium containing 0.5 mg/mL of MTT, and the incubation was continued for 2 h. At the end of incubation, medium containing MTT was removed, and insoluble formazan crystals formed in cells were dissolved in 150 μL of dimethyl sulfoxide (Sigma). The absorbance was measured at 570 nm using 96-well plate FLUOstar Optima [9].

### 3.13. Statistical Analysis

Data were presented as means of values from three independent experiments ± standard deviation (SD). Data from multiple groups were analyzed using the one-way variance assay followed with *post-hoc* tests. Differences at *p* < 0.05 were considered as significant.

## 4. Conclusions

The present study for the first time demonstrated C3G neutralized the effects of diabetes-associated glyLDL on NOX activation, the upregulation of RAGE, mitochondrial dysfunction and impaired cell viability in cultured vascular EC. The findings may help to design novel nutraceutical therapy to reduce risks of cardiovascular complications in diabetic animal model and patients.
